# Low Phytoestrogen Levels in Feed Increase Fetal Serum Estradiol Resulting in the “Fetal Estrogenization Syndrome” and Obesity in CD-1 Mice

**DOI:** 10.1289/ehp.10448

**Published:** 2007-11-26

**Authors:** Rachel L. Ruhlen, Kembra L. Howdeshell, Jiude Mao, Julia A. Taylor, Franklin H. Bronson, Retha R. Newbold, Wade V. Welshons, Frederick S. vom Saal

**Affiliations:** 1 Division of Biological Sciences, University of Missouri-Columbia, Columbia, Missouri, USA; 2 Department of Zoology, University of Texas at Austin, Austin, Texas, USA; 3 Developmental Endocrinology and Endocrine Disruptor Section, Laboratory of Molecular Toxicology, National Institute of Environmental Health Sciences, National Institutes of Health, Department of Health and Human Services, Research Triangle Park, North Carolina, USA; 4 Department of Biomedical Sciences, University of Missouri-Columbia, Columbia, Missouri, USA

**Keywords:** casein, estradiol, fat, glucose tolerance, leptin, metabolic syndrome, obesity, puberty, reproductive organs, soy

## Abstract

**Background:**

Although estrogenic chemicals can disrupt development of the reproductive system, there is debate about whether phytoestrogens in soy are beneficial, benign, or harmful.

**Objectives:**

We compared reproductive and metabolic characteristics in male and female mice reared and maintained on non-soy low-phytoestrogen feed or soy-based high-phytoestrogen feed.

**Methods:**

The low-phytoestrogen diet was non-soy PMI 5K96 (verified casein diet), and the high-phytoestrogen diet consisted of soy-based PMI 5008 during pregnancy and lactation and soy-based PMI 5001 maintenance feed after weaning.

**Results:**

In fetuses whose mothers consumed the low-phytoestrogen PMI 5K96 feed, we found a paradoxical significant elevation in endogenous serum estradiol, which was associated postnatally with adverse reproductive outcomes referred to as the “fetal estrogenization syndrome (FES)”. In females, this syndrome included early puberty and increased uterine responsiveness to estrogen, and in males, it included reduced testis, epididymis, and seminal vesicle size, but an enlarged prostate. The low-phytoestrogen–fed males and females were lighter at birth, but, between weaning and adulthood, they became obese and developed abnormally high serum leptin levels; these males, but not females, showed impaired glucose regulation.

**Conclusions:**

Removing phytoestrogens from mouse feed produces an obese phenotype consistent with metabolic syndrome, and the associated reproductive system abnormalities are consistent with FES due to elevated endogenous fetal estradiol. Laboratory rodents may have become adapted to high-phytoestrogen intake over many generations of being fed soy-based commercial feed; removing all phytoestrogens from feed leads to alterations that could disrupt many types of biomedical research.

The typical view of estrogen is that it is associated with a reduction in food intake, fat deposition, and body weight in adults (Cooke and Naaz 2004). However, a maxim in developmental biology and pediatric medicine is that it is inappropriate to use effects in adults to predict effects during development (i.e., babies are not little adults) (National Research Council 1993). During critical periods in development, estrogenic drugs and chemicals can have unexpected stimulatory effects on the differentiation of adipocytes, as well as on postnatal growth and obesity (Cooke and Naaz 2004; Howdeshell et al. 1999; Newbold et al. 2005). There is also an extensive literature showing that administration of supplementary estrogen to rodents, even at very low doses, disrupts normal development of the reproductive system (Welshons et al. 2003, 2006). The administration of diethylstilbestrol (DES) to millions of women, and experiments in mice showing a high concordance in pathologic outcome, revealed that mice are appropriate sentinels for studies of the effects of estrogen on development in humans (Newbold 1995; Swan and vom Saal 2001).

The prediction that obesity and other abnormalities are related to events (e.g., alterations in fetal hormone levels due to maternal stress, maternal nutrition, or exposure to environmental chemicals) that occur during fetal development is know as the “fetal basis of adult disease” hypothesis. For example, there is now considerable evidence that metabolic syndrome in humans is related to factors that influence growth *in utero.* Specifically, intrauterine growth restriction (IUGR) that is followed by a high velocity of postnatal growth results in adult obesity and associated metabolic diseases. The “thrifty phenotype” hypothesis predicts that this is due to the inability of the IUGR individual’s homeostatic systems that regulate body weight to function when there is a high level of energy intake common in developed countries (Wild and Byrne 2004; Yajnik 2001). Endocrine changes are associated with obesity (for example, there is an increase in the hormone leptin that is produced by adipocytes), and they not only impact the neuroendocrine control of metabolic systems and appetite (Kalra et al. 2005; Schwartz 2001), but they also impact reproductive tissues and processes (Baratta 2002).

Although estrogenic drugs such as DES and estrogenic environmental chemicals such as bisphenol A (BPA) can profoundly disrupt normal development at very low doses in rats and mice (vom Saal and Welshons 2006; Welshons et al. 2006), there remains much controversy as to whether estrogenic chemicals that are synthesized in plants are harmful or beneficial. One basis for this controversy is related to the issue of selective estrogen receptor modulators—estrogens whose effects can differ markedly in different tissues and at different times in life (Welshons et al. 2006). Soy is the major source of plant-phytoestrogen exposure in humans and in laboratory rodents, which have been bred and maintained by commercial animal breeders for many generations on soy-containing feed. This suggests that laboratory rodents have been subjected to selection for traits that allow them to tolerate relatively high levels of phytoestrogens present in the most commonly used commercial soy-based feeds.

In the present study we compared mice reared and maintained on a low- (non-soy) and high-phytoestrogen–containing soy-based feed. The objective was to determine the effects on phenotype of eliminating soy phytoestrogens from feed, because all of our prior research involving the effects of endocrine-disrupting chemicals has been conducted with animals maintained on soy-based Purina-Mills (PMI; St. Louis, MO) feed. The basis for our interest in this issue was a desire to avoid the potential disruption of experiments concerning the effects of estrogenic endocrine-disrupting chemicals because of the marked variability in phytoestrogen levels known to exist from one batch of soy to another (Heindel and vom Saal 2008). Many environmental factors influence phytoestrogen levels per gram of soy protein, and diets with a constant level of soy protein can show variable levels of phytoestrogens that are great enough to disrupt studies of the effects of DES in mice (Thigpen et al. 2003). One logical assumption would thus be to eliminate this problem by using a soy-free feed.

In contrast to our prediction, we found that there is a paradoxical estrogenization of fetuses that occurs when pregnant female mice are fed a low-phytoestrogen, soy-free feed because of an elevation in endogenous fetal serum estradiol; this is related to adverse postnatal reproductive traits similar to those produced by exposing fetuses to estradiol, DES, and other man-made estrogenic chemicals such as BPA. In addition, the low-phytoestrogen feed produced a phenotype consistent with metabolic syndrome. Our findings suggest a dramatic difference in phenotype of mice exposed to plant phytoestrogens compared with estradiol or man-made estrogenic drugs and chemicals, which is consistent with other recent findings (Dolinoy et al. 2007).

## General Methods

### Experimental animals and feeds

CD-1 mice (*Mus musculus domesticus*) were purchased from Charles River (Wilmington, MA) and maintained as an outbred colony with periodic replacement of breeder males and females. Animals were housed in standard polypropylene mouse cages on corncob bedding. Glass water bottles were used, and water was purified by reverse osmosis and carbon filtration. Rooms were kept at 23°C and maintained on a 12-hr light and 12-hr dark cycle, with lights on at 1100 hours so that timed matings could occur at the end of the dark phase of the cycle. All animal procedures were approved by the University of Missouri Animal Care and Use Committee and conform to the NIH *Guide for the Care and Use of Laboratory Animals* (Institute of Laboratory Animal Resources 1996). The animals were treated humanely and with regard for alleviation of suffering.

We randomly selected adult female CD-1 mice from our colony (previously maintained after weaning on PMI 5001) to be placed on either PMI 5K96 or PMI 5008 for 7 days, after which they were paired with males until they were visibly pregnant. Females remained on these diets throughout pregnancy and lactation. The number, weight, and sex of pups were recorded on the day of birth. At weaning on postnatal day (PND) 20, pups reared on the PMI 5008 breeder chow were switched to PMI 5001 maintenance chow, which has less fat and metabolizable energy. The combined use of PMI 5008/5001 is standard in many laboratories. Pups reared on PMI 5K96 feed remained on this feed after weaning. These procedures for administering feeds were used in all experiments, although the age at weaning differed in a few experiments. The PMI 5K96 feed does not contain soy, and casein is one of the sources of protein in this feed; Purina-Mills describes PMI 5K96 as a “verified casein diet” (LabDiet 2007). In contrast, the PMI 5008 and PMI 5001 feeds are soy based and thus contain soy isoflavones. The components of these different feeds are provided in Table 1.

For postnatal studies, we weaned 18 for the PMI 5008/5001 diet and 19 for the PMI 5K96 diet. Average litter size at birth was 13 pups, and there was no effect of feed on litter size. At weaning, males and females were separated and housed three or four per cage unless otherwise noted.

### Estradiol radioimmunoassay

The estradiol radioimmunoassay was performed as previously described (vom Saal et al. 1990). Briefly, [^125^I]estradiol and antisera were obtained from ICN Biomedicals (Costa Mesa, CA), and unlabeled estradiol was obtained from Steraloids (Wilton, NH). Sensitivity of the assay was 0.5 pg/tube. Intraassay and interassay coefficients of variation were 5% and 4%, respectively. We determined the percent cross-reactivity of the estradiol antiserum with estrone to be 0.6%. Cross-reactivity with other steroids was reported by ICN to be negligible.

### Leptin radioimmunoassay

Leptin levels were measured in serum by radioimmunoassay (Linco leptin assay kit) according to the manufacturer’s protocol (Linco Research Immunoassay, St. Charles, MO).

### Statistical procedures

All organ weight data were first analyzed by analysis of covariance (ANCOVA) to determine whether organ weight measures needed to be corrected for body weight. If body weight accounted for a significant component of the variance, then group means were adjusted for body weight. If body weight did not account for a significant component of the variance for a specific measure, then the data were reanalyzed by analysis of variance (ANOVA). With the exception of the seminal vesicles, all other organs examined were significantly correlated with body weight, and these organ weight data were analyzed by ANCOVA rather than ANOVA, with body weight as the covariate. If more than one animal per litter was used in an experiment, litter was included as a main effect variable and the *F*-value for the diet comparison was divided by the *F*-value for litter. We conducted planned comparisons using the LSmeans test (SAS Institute Inc., Cary, NC) on results if the overall ANOVA or ANCOVA was statistically significant. Results were considered statistically significant at *p* < 0.05. All data are presented as the mean and SE.

## Experimental Methods and Results

### MCF-7 human breast cancer cell bioassay to determine estrogenic activity in feed

We measured the estrogenic activity in the feed by solvent (methanol) extraction and examination of the degree to which the extract stimulated proliferation of human MCF-7 breast cancer cells; this bioassay provides a highly sensitive method for detecting estrogenic activity in feed (Welshons et al. 1990, 2003). Use of this bioassay to screen feeds for estrogenic activity prior to use is necessary because there can be significant batch-to-batch variability in soy isoflavones in soy-based feeds, as well as variability in other estrogenic contaminants in feeds that do not contain soy. Briefly, approximately 10,000 cells were seeded per well of a 24-well plate on day 0 in phenol red-free (estrogen-free) medium, fed on day 1 with the same medium, and then treated on days 3–6 with the test media, with daily medium changes. Test media for feeds contained methanol extracts from feed. On day 7 the wells were washed twice with 1 mL Hanks’ balanced salt solution, and each well was then assayed for DNA content.

Total estrogenic activity is expressed as parts per million equivalents of a reference, genistein, which is the primary phytoestrogen in soy, even though the total estrogenic activity is due to other estrogenic compounds in addition to genistein. The standard curve was based on triplicate assays, and extracted samples were each added to three wells containing cells. When apparent estrogenic activity was observed, the sample was run a second time to confirm the initial results and to examine whether proliferation occurred with and without addition of an antiestrogen (ICI 182,780) to confirm an estrogenic mechanism for stimulation of proliferation. In all samples tested in this experiment, the antiestrogen inhibited all proliferation observed above estrogen-free controls that did not contain any extract from feed.

We found very low estrogenic activity for the batch of PMI 5K96 feed (expressed as genistein equivalent units; 3.9 ppm) compared with the batches of PMI 5008 (40.0 ppm) and PMI 5001 (25.8 ppm). Because the PMI 5K96 feed is not completely free of estrogenic activity, it is referred to as a low-phytoestrogen feed, although the source of the estrogenic activity was not identified.

### Serum estradiol in pregnant females and fetuses

A separate group of 13 females fed PMI 5K96 and 10 females fed PMI 5008 were killed on gestation day (GD) 18 for analysis of serum estradiol levels. These females were paired with stud males for 4 hr each day at the end of the dark phase of the light:dark cycle and then examined for vaginal plugs, which was GD0. Females from each feed group were housed three per cage, and fetuses were delivered on GD18 (1 day before parturition). Maternal and fetal blood samples were collected for measurement of estradiol. Blood from males and from females within each litter was pooled, with each litter yielding one value for male and one value for female fetuses.

**Figure 1:**
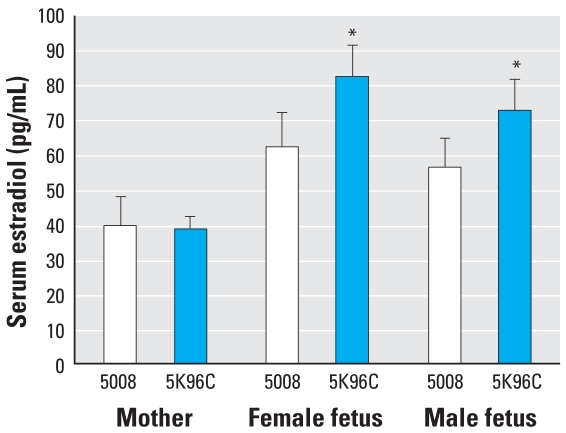
Mean (+ SE) serum estradiol (pg/mL) on GD18 in pregnant females and fetuses. Blood from males and from females within each PMI 5K96C litter (*n* = 13) and PMI 5008 litter (*n* = 10) was pooled. *p < 0.05.

On GD18 (1 day before parturition and during differentiation of pre-adipocytes and the reproductive system), serum estradiol levels in fetuses whose mothers were fed PMI 5K96 feed were significantly elevated relative to levels in the fetuses whose mothers were maintained on PMI 5008 feed (Figure 1). We found no differences in serum estradiol levels in the pregnant females on the PMI 5008 and PMI 5K96 feeds, suggesting that the feed affected estradiol production by the fetuses, not the mothers. The PMI 5K96 feed, with little estrogenic activity and casein rather than soy protein with isoflavones, thus had the paradoxical effect of increasing exposure of mouse fetuses to the potent endogenous estrogen estradiol.

### Postnatal body weight, abdominal fat, and serum leptin

**Figure 2:**
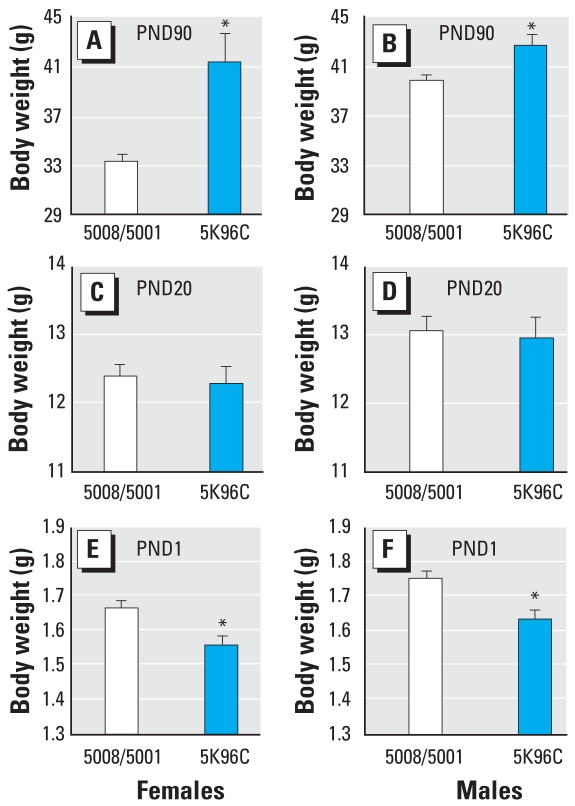
Mean (+ SE) body weight of female pups (*A,C,E; n* = 18 PMI 5008/5001; *n* = 19 PMI 5K96C) and male pups (*B,D,F; n* = 18 PMI 5008/5001; *n* = 19 PMI 5K96C) at birth (PND1; *E,F*), weaning (PND20; *C,D*), and adulthood (PND90; *A,B*). *p < 0.01.

To determine the rate of postnatal growth, we weighed one randomly selected male and female from each litter (identified by a toe clip) at birth and weaning and in adulthood; weighing pups every day alters their postnatal growth (Ruhlen RL, unpublished data). Pups produced by PMI 5008-fed females were significantly heavier at birth than pups produced by PMI 5K96-fed females (Figure 2). There was no effect of feed on body weight on PND20 (weaning), whereas on PND90 (adulthood), the PMI 5K96-fed males and females were significantly heavier than PMI 5008/5001-fed animals of the same sex; adult males fed PMI 5K96 weighed 11% more than males fed PMI 5008/5001, and females fed PMI 5K96 weighed 27% more than females fed PMI 5008/5001. There was thus a reversal of the effect of feed type on prenatal growth, which was significantly increased by high-phytoestrogen–containing feed, and post-weaning growth, which was reduced by high-phytoestrogen–containing feed. Males fed PMI 5008/5001, but not PMI 5K96, were significantly heavier than females on the same feed.

**Figure 3:**
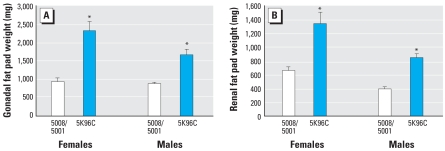
Mean (+ SE) weight of gonadal (*A*) and renal (*B*) fat pads from 3-month-old males and females (PMI 5008/5001, *n* = 10; PMI 5K96C, *n* = 9). *p < 0.01.

On PND90 we examined the fat pads associated with the gonads (epididymal fat pads in males and parametrial fat pads in females) and with the kidneys (renal fat pads) in males and females, which together represent abdominal fat in mice. Mice fed PMI 5K96 throughout life were obese compared with those fed PMI 5008/5001 (Figure 3). Gonadal and renal fat pads from males fed PMI 5K96 weighed 93% and 115%, respectively, more than those of males fed PMI 5008/5001. Gonadal and renal fat pads from females fed PMI 5K96 weighed 126% and 86%, respectively, more than those of females fed PMI 5008/5001.

**Figure 4:**
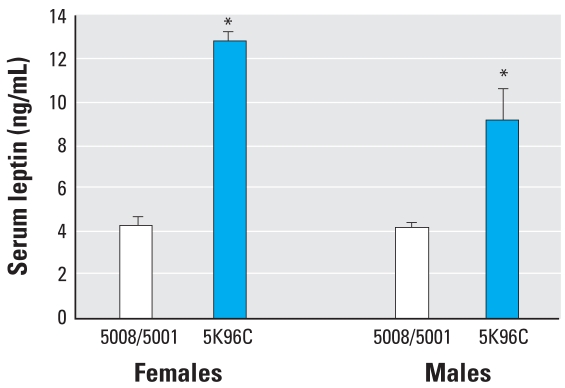
Mean (+ SE) serum leptin from 3-month-old males (*n* = 7/group) and females (PMI 5008/5001, *n* = 10; PMI 5K96C, *n* = 9). *p < 0.01.

Serum leptin was increased 121% in males and 174% in females fed PMI 5K96 compared with males and females fed PMI 5008/5001 (Figure 4). Leptin is produced by adipocytes, and serum leptin was significantly correlated (*r* = 0.79; *p* < 0.001) with total weight of fat pads.

### Adult glucose tolerance

We examined glucose tolerance as previously described (Heine et al. 2000). The same animals that were examined for body weight, fat-pad weight, and serum leptin were examined in this study, which was conducted 1 week before fat pad collection on PND90. Animals were fasted overnight (12–14 hr), and injected intra-peritoneally with 2 mg glucose per gram body weight in 0.9% saline solution. Blood glucose was measured with an Accu-Chek Glucometer (Roche, Indianapolis, IN) by tail nick. Linearity of the glucometer was verified with the Accu-Chek Linearity Test Kit (Roche).

**Figure 5:**
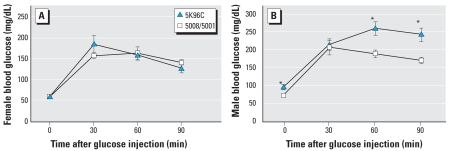
Mean (± SE) blood glucose before (time 0) and 30, 60, and 90 min after an intraperitoneal injection of 2 mg/g glucose (in saline) in females (*A*) and males (*B; n* = 10/group) 1 week before sacrifice for examination of body fat and serum leptin on PND90. *p < 0.01.

Basal blood glucose levels and glucose clearance after a glucose injection were not different based on type of feed in females. In contrast, males fed PMI 5K96 had impaired glucose clearance compared with males fed PMI 5008/5001 (Figure 5). Specifically, compared with males fed PMI 5008/5001, basal blood glucose was significantly elevated in males fed PMI 5K96 (*p* < 0.001); at both 60 and 90 min postglucose injection, blood glucose was significantly higher in PMI 5K96-fed males than in PMI 5008/5001-fed males.

### Uterine response to estradiol and serum leptin in prepubertal females

The prepubertal uterotrophic assay has been shown to be influenced by exposure to estrogen during fetal–neonatal development in mice, with developmental exposure to low doses of estrogen increasing uterine responsiveness to estrogen (Newbold et al. 2004). In addition, the timing of puberty in female mice can be accelerated by females being maintained on a diet with very high estrogenic activity after weaning (Thigpen et al. 2003). This experiment thus provides a means of determining whether the uterine response to estradiol would be enhanced in females being fed the soy-based PMI 5001 feed containing high levels of phytoestrogens or PMI 5K96 with low-phytoestrogen levels that had resulted in elevated endogenous estradiol levels during fetal life.

Two randomly selected females per litter were weaned on PND17. The females were anesthetized with isoflurane and implanted subcutaneously with a Silastic capsule (0.62 i.d., 1.25 o.d.) that was 5 mm long between the capped ends. One female from each litter received a capsule contained 0.25 μg 17β-estradiol dissolved in 10 μL tocopherol-stripped corn oil (Fisher Scientific, Pittsburgh, PA). This dose of estradiol results in about 80% of the maximum uterine growth response that can be induced with estradiol at this age. The other females from the same litters (controls) were implanted with capsules containing only oil vehicle. The capsules had been preincubated for 24 hr in a physiologic phosphate-buffered saline solution with albumin added to stabilize the rate of estradiol release, which is then constant for the 3 days of this experiment.

**Figure 6:**
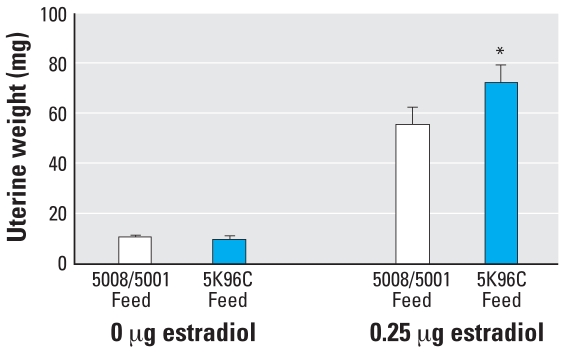
Mean (+ SE) uterine weight on PND20 in response to a low dose of estradiol or oil vehicle via a subcutaneous Silastic implant (*n* = 10/group). *p < 0.05.

When examined on PND20, uterine wet weight in females treated with the oil vehicle did not differ based on diet. However, the uteri of females fed PMI 5K96 were significantly (*p* < 0.05) heavier in response to estradiol stimulation compared with those of females fed PMI 5008/5001 (Figure 6). Body weight was slightly, but not significantly, higher in females fed PMI 5K96 (10.50 ± 0.49 g) relative to females fed PMI 5008/5001 (9.59 ± 0.45 g; *p* > 0.1). Neither feed nor estradiol treatment influenced serum leptin, which was about 2.2 ng/ml.

### Body weight, uterine weight, histology, and serum leptin on PND26

In this experiment, 10 females from each diet group were weaned and weighed on PND19 and housed two or three per cage; as in other experiments, the PMI 5008 feed was replaced with PMI 5001. On PND26 (around the time that puberty would begin) the females were euthanized; blood was collected for analysis of serum leptin; body weight and uterine wet weight were measured; and uteri were fixed for histologic analysis of luminal epithelial cell height, which was measured using Image Pro software (Media Cybernetics, Bethesda, MD). These uterine assays serve as biomarkers of whether females were experiencing an increase in estrogen, indicating that puberty had begun.

Longitudinal sections (7 μm) of the right half of the uterus were mounted on poly-l-lysein–coated glass slides stained with hematoxylin and eosin and examined using an Olympus BH-2 light microscope (Olympus Corp., New Hyde Park, NY) with Image Pro. Uterine luminal epithelial cell height was determined by calculating the average of three measurements of randomly selected cells at three nonadjacent sections in one uterine horn.

**Figure 7:**
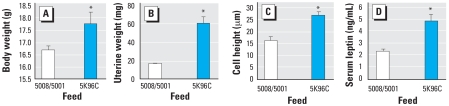
Mean (+ SE) body weight (*A*), uterine weight (*B*), height of the uterine luminal epithelium (*C*), and serum leptin (*D*) in females weaned on PND19 and examined on PND26. *p < 0.01.

At weaning on PND19, the body weight of PMI 5008 females was 10.0 ± 0.2 g and that of PMI 5K96 females was 10.1 ± 0.3 g. Compared with females on the PMI 5008/5001 diets, PND26 females fed PMI 5K96 were significantly heavier and had significantly enlarged uteri, increased epithelial cell height, and significantly elevated serum leptin. Thus, after consuming PMI 5K96 feed for 7 days after weaning, mice fed PMI 5K96 had accelerated body growth and increased serum leptin (suggesting an increase in body fat). The PMI 5K96 feed stimulated an increase in both uterine wet weight and epithelial cell height, indicative of accelerated puberty in the PMI 5K96-fed females compared with females on the high-phytoestrogen PMI 5008/5001 feed (Figure 7).

### Onset of fertility in females

Two randomly selected females per litter were weaned on PND20 and paired with a sexually experienced male and monitored for the age at parturition. When the female was visibly pregnant, the male was removed. We recorded the age of the dam at delivery and the number, body weights, and sex ratio of pups. This procedure provides a direct measure of the timing of onset of fertility (the first ovulation and mating) as well as reproductive capacity in peripubertal female mice.

The females fed PMI 5K96 produced pups at a significantly younger age (44.7 ± 0.3 days) than the females fed PMI 5008/5001 (46.5 ± 0.6 days; *p* = 0.01). We found no significant difference in the number of pups produced by females on either feed, although the females fed PMI 5K96 produced slightly more pups per litter, and as a result, the litters tended to weigh more (*p* = 0.06).

### Adult male reproductive organs

**Figure 8:**
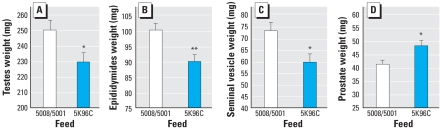
Mean (+ SE) wet weight of testes (*A*), epidydimides (*B*), seminal vesicles (*C*), and prostates (*D*) collected from 3-month-old male mice fed PMI 5K96C (*n* = 24) or PMI 5008/5001 (*n* = 22). With the exception of the seminal vesicles, comparisons are based on ANCOVA, because for each organ measured, body weight accounted for a significant component of the variance. *p < 0.05. **p < 0.01.

On PND90, we collected reproductive organs from male mice fed PMI 5K96 (*n* = 24) or PMI 5008/5001 diets (*n* = 22). All male reproductive organs were significantly different based on the type of feed (Figure 8). The weights of the testes and Wolffian duct–derived organs (epididymides and seminal vesicles) were significantly smaller in males fed PMI 5K96 than in males fed PMI 5008/5001. In contrast, for the prostate, which differentiates from the urogenital sinus, males fed PMI 5K96 had heavier prostates than those fed PMI 5008/5001. In addition to reproductive organs, males on the PMI 5K96 feed had a smaller liver (2.08 ± 0.03 g) and a smaller right kidney (298.9 ± 6.4 mg) than PMI 5008/5001 males (liver: 2.22 ± 0.03 g, *p* < 0.05; kidney: 354.7 ± 6.7 mg, *p* < 0.05).

## Discussion

A paradoxical finding in the present study was that the low-phytoestrogen PMI 5K96 feed resulted in a significant elevation in fetal serum estradiol by 24% in males and 37% in females compared with male and female fetuses from dams on the high-phytoestrogen soy PMI 5008 feed. We previously reported that administration of estradiol to pregnant mice that led to a similar elevation in fetal serum estradiol, and also maternal administration of low doses of estrogenic drugs, produced an increase in prostate gland number and size in male offspring maintained on PMI 5008/5001 feed (Timms et al. 2005; vom Saal et al. 1997). The finding that adult males fed PMI 5K96 had enlarged prostates compared to PMI 5008/5001-fed males is thus consistent with these earlier findings, as well as other reports that isoflavones are associated with a reduction in prostate size (Jensen and Ritskes-Hoitinga 2007).

**Figure 9:**
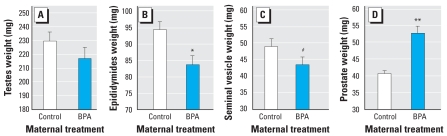
Mean (+ SE) weight of reproductive organs in adult CF-1 male mice as a result of fetal exposure to 2 μg/kg/day BPA. BPA or oil vehicle (controls) was fed to pregnant females on GD11–GD17. All pregnant and lactating females were fed PMI 5008 and all males were fed 5001 after weaning. The effects of BPA on the reproductive organs in male mice maintained on the PMI 5008/5001 feed was identical to the effects of the PMI 5K96C feed compared with PMI 5008/5001 feed shown in Figure 8. This suggests that the elevation in fetal serum estradiol caused by the PMI 5K96C feed (Figure 1) produced a permanent effect on the reproductive organs in males, similar to the permanent effect caused by fetal exposure to BPA. Adapted from vom Saal et al. (1998). *p < 0.05. **p < 0.01. ^#^*p* = 0.08.

We also previously reported that feeding pregnant mice a low dose of BPA, the estrogenic chemical used to make polycarbonate plastic, resulted in exactly the same pattern of reproductive organ differences in male offspring when examined in adulthood (small testes, epididymides, and seminal vesicles, but enlarged prostate) compared with untreated males (vom Saal et al. 1998); this experiment was also conducted with PMI 5008/5001-fed male mice (Figure 9). Fetal exposure to a relatively small increase in estradiol or man-made estrogenic chemicals in the blood thus permanently decreases testis, epididymis, and seminal vesicle weight but through different mechanisms increases prostate size (Nonneman et al. 1992; Richter et al. 2007; Timms et al. 2005; vom Saal 1989; vom Saal et al. 1997).

In females, exposure to estrogenic chemicals such as BPA during fetal development leads to an accelerated postnatal growth and accelerated onset of puberty (Howdeshell et al. 1999). In the present study we found that on PND26, females on PMI 5K96 showed accelerated growth, elevated serum leptin, and uteri that were being stimulated by estrogen (indicative of the onset of puberty), whereas the uteri of PMI 5008/5001 females did not show evidence of estrogen stimulation, and thus were still prepubertal. In addition, the first ovulation resulting in pregnancy, marking the end of puberty and onset of fertility, occurred earlier in females fed PMI 5K96 than in females fed PMI 5008/5001. Females fed PMI 5K96 also showed a greater increase in uterine weight in response to stimulation by estradiol in the prepubertal uterotrophic assay, which is also consistent with developmental exposure to estrogenic chemicals (Newbold et al. 2004). Taken together, these findings suggest a permanent adverse consequence on the reproductive system in male and female offspring whose mothers ate the low-phytoestrogen PMI 5K96 feed resulting from the elevated estradiol levels during fetal life [the fetal estrogenization syndrome (FES)].

Although there is some evidence that soy isoflavones can reduce activity of aromatase, the enzyme that metabolizes androgen to estrogen (Adlercreutz et al. 1993), preliminary studies (vom Saal FS, unpublished data) suggest that the lower estradiol in high-phytoestrogen–exposed fetuses is related to a reduction in production of the estrogen-binding plasma protein alphafetoprotein (AFP) in the fetal liver rather than a decrease in production of aromatase (primarily present in the fetal mouse brain). We previously reported that the percent of total free serum estradiol is constant over a wide range of total serum estradiol concentrations in rat and mouse serum (Montano et al. 1995). This finding suggests that the difference in total serum estradiol in fetuses whose mothers were fed low- and high-phytoestrogen–containing feed, which may be due to a difference in plasma AFP levels, would result in a proportional difference in free, biologically active serum estradiol; this represents about 0.2–0.3% of total serum estradiol in mouse and rat fetuses (Greenstein 1992; Montano et al. 1995; vom Saal et al. 1997).

There has been considerable interest in the hormonal activity of diets that contain soy phytoestrogens with regard to the potential for adverse effects in infants (Newbold et al. 2001; Sheehan 1997). Against the background of very low endogenous estradiol found in females between birth and the onset of puberty, a phytoestrogen-rich diet can stimulate uterine growth and accelerate puberty (Boettger-Tong et al. 1998; Thigpen et al. 2003), and administration of genistein at amounts in soy infant formula to neonatal mice, against a very low background level of estradiol, caused uterine cancer in midlife, similar to effects of the potent estrogenic drug DES (Newbold et al. 2001). In contrast, phytoestrogens can have antagonistic effects on estradiol when there are very high levels of estradiol, such as during pregnancy (Adlercreutz et al. 1993). There is now extensive evidence that chemicals classified as estrogens can have markedly different activities at different times in life, in relation to different background levels of endogenous estradiol, and different activities in different tissues. Thus, chemicals such as the drug tamoxifen, phytoestrogens in soy, and BPA in plastic are referred to as selective estrogen receptor modulators (Welshons et al. 2006).

Our findings suggest that the high-phytoestrogen content of PMI 5008 feed resulted in a decrease in fetal serum estradiol relative to the low-phytoestrogen PMI 5K96 feed. A soy-based diet can thus have significant beneficial effects on phenotype in mice by virtue of reducing fetal serum estradiol, which is protective against the FES. This finding emphasizes that the simplistic assumption that a diet with phytoestrogens will estrogenize fetuses is not valid with regard to fetal development. In contrast, this assumption is clearly correct between birth and weaning when endogenous estrogen is very low; lactation is the only time in life when mammals have evolved to live on milk, with casein as the sole source of protein and thus very low exposure to phytoestrogens regardless of phytoestrogen levels in the maternal diet (Doerge et al. 2006; Franke and Custer 1996). Taken together, these findings suggest that feeds with different levels of phytoestrogens may be desirable for use during pregnancy and lactation as opposed to after weaning, whereas for studies that involve maintaining female rodents after the midlife cessation of ovulation, a different diet composition may be most appropriate. Clearly, this relatively unstudied area represents an important research need (Heindel and vom Saal 2008).

We found that the low-phytoestrogen PMI 5K96 feed resulted in prediabetic traits of excess fat and impaired glucose tolerance relative to the phytoestrogen-rich PMI 5008/5001 feeds, even though all of these diets provide very similar calories per gram (Table 1). Metabolic syndrome refers to the clustering of risk factors for cardiovascular disease and type 2 diabetes, such as impaired glucose metabolism and insulin resistance, obesity, and hypertension (Kahn et al. 2001; Liese et al. 1998). The increasing incidence of these conditions in the United States and other countries has generated attention on potential causes, specifically on events *in utero* that are associated with premature mortality from these diseases. The PMI 5K96-fed mothers produced fetuses that were significantly lighter at birth relative to pups produced by soy-fed mothers, but by shortly after weaning this relationship was reversed, and by adulthood the animals on the low-phytoestrogen feed had become obese.

Additional evidence in support of the hypothesis that mice fed a low-phytoestrogen diet throughout life have metabolic syndrome is provided in another related study in which CD-1 mice were fed Harlan 8604 soy-based feed with high-phytoestrogen levels or a soy-free purified diet by Zeigler (Cederroth et al. 2007). These authors reported that, similar to our findings, the mice administered the feed without soy isoflavones were fatter and showed impaired glucose tolerance, as well as impaired resistance to cold and reduced activity levels (Cederroth et al. 2007).

## Conclusions

Additional research will be needed to determine whether all of these outcomes are caused by elevated estradiol levels during fetal life. However, every one of our experiments concerning reproductive end points provided a comparison between the potential for high estrogenic activity in soy-based feed to stimulate estrogenic responses at the time of data collection in postnatal life (Thigpen et al. 2003) compared with the predicted outcome of elevated fetal estradiol on the outcome based on a large number of published studies on this subject. Our results did not indicate that the soy phytoestrogens in PMI 5001 feed after weaning had an effect on the outcome of any experiment, although the findings were consistent with the outcome predicted as a result of increasing estrogen levels during fetal life on the reproductive system.

The “fetal basis of adult disease” hypothesis proposes that endocrine or other metabolic disturbances in the fetus can lead to development of a phenotype consistent with metabolic syndrome. The prediction that developmental exposure to estradiol is involved in the increase in postnatal body weight in PMI 5K96-fed mice, and is thus a newly discovered component of the FES, is supported by findings that neonatal exposure to a low dose of DES significantly increased adult body weight relative to untreated mice in an experiment in which the mice were maintained on a feed containing phytoestrogens (Newbold et al. 2005). Reproductive problems associated with diabetes and obesity have generally been assumed to be secondary to these metabolic disorders.

In summary, our findings suggest the possibility of a common etiology for fetal effects on adult obesity and reproductive disorders, because all of the findings concerning the effects of these high- and low-phytoestrogen–containing feeds are consistent with the hypothesis that the elevated levels of estradiol in fetal mice whose mothers consumed a low-phytoestrogen feed produced the suite of adverse reproductive outcomes referred to as FES.

## Figures and Tables

**Table 1:** Percent components of soy-based PMI 5001 and 5008 feeds and non-soy PMI 5K96 feed and components of these feeds that are present (+) or absent (–) in each type of feed.

	PMI 5008	PMI 5001	PMI 5K96
Components of feeds			
Percent protein	23.5	23.4	18.9
Percent fat (ether extract)	6.5	4.5	4.3
Percent fiber (crude)	3.8	5.3	3.6
Percent starch	34.9	31.9	44.8
Percent sucrose	2.6	3.7	0.4
Total digestible nutrients (%)	81.2	76	75.2
Gross energy (kcal/g)	4.15	4	4.05
Physiological fuel value (kcal/g)	3.5	3.3	3.44
Metabolizable energy (kcal/g)	3.31	3.04	3.15
Differences between feeds			
Animal fat preserved with BHA	+	+	−
Biotin	−	−	+
Cane molasses	+	+	−
Casein	−	−	+
Corn gluten meal	−	−	+
Corn oil	−	−	+
Dehydrated alfalfa meal	+	+	−
Dicalcium phosphate	−	−	+
Dried beet pulp	+	+	−
Dried whey	+	+	−
Ferrous carbonate	+	+	−
Ferrous sulfate	−	+	−
Ground wheat	+	−	+
Magnesium oxide	−	−	+
Nicotinic acid	−	+	−
Porcine meat meal	+	+	−
MSB (vitamin K)	+	−	+
Nicotinic acid	+	−	+

MSB, menadione sodium bisulfite (source of vitamin K). Additional ingredients found in all diets: brewers dried yeast, calcium carbonate, calcium iodate, calcium pantothenate, cholecalciferol, choline chloride, cobalt carbonate, copper sulfate, cyanocobalamin, dehulled soybean meal, dl-alpha tocopheryl acetate, DLmethionine, fish meal, folic acid, ground corn, ground oats, manganous oxide, pyridoxine hydrochloride, riboflavin, salt, thiamin mononitrate, vitamin A acetate, wheat middlings, zinc oxide, and zinc sulfate. Data from Labdiet (2007).
